# Impact of coronary artery bypass surgery and percutaneous coronary intervention on mortality in patients with chronic kidney disease and on dialysis: A systematic review and meta-analysis

**DOI:** 10.1097/MD.0000000000004129

**Published:** 2016-07-08

**Authors:** Pravesh Kumar Bundhun, Akash Bhurtu, Meng-Hua Chen

**Affiliations:** Institute of Cardiovascular Diseases, the First Affiliated Hospital of Guangxi Medical University, Nanning, Guangxi, P.R. China.

**Keywords:** chronic kidney disease, coronary artery bypass surgery, dialysis, end-stage renal disease, mortality, percutaneous coronary intervention

## Abstract

Controversies have been observed among previously published and recently published studies comparing coronary artery bypass surgery (CABG) and percutaneous coronary intervention (PCI) in patients with chronic kidney disease (CKD) and patients on chronic dialysis. This study aimed to show the impact of CABG and PCI on mortality in these patients.

Electronic databases were searched for studies comparing CABG and PCI in patients with CKD. The primary outcome was all-cause death whereas the secondary endpoints included other adverse cardiovascular outcomes reported. Causes of death were also analyzed. Odds ratios (ORs) with 95% confidence intervals (CIs) were used to express the pooled effect on discontinuous variables and the pooled analyses were performed with RevMan 5.3.

Eighteen studies involving a total number of 69,456 patients (29,239 patients in the CABG group and 40,217 patients in the PCI group) were included in this meta-analysis. Short-term mortality insignificantly favored PCI with OR: 1.24, 95% CI: 0.93–1.65; *P* = 0.15. Mortality at 1 year was similar in both groups with OR: 0.99, 95% CI: 0.91–1.08; *P* = 0.86, whereas the long-term mortality significantly favored CABG in patients with CKD and in patients on chronic dialysis with OR: 0.81, 95% CI: 0.70–0.94; *P* = 0.007 and OR: 0.81, 95% CI: 0.69–0.96; *P* = 0.01, respectively.

In patients with CKD, the impact of CABG on the short-term mortality was insignificantly higher compared to PCI whereas at 1 year, a similar impact was observed. However, the impact of PCI on mortality was significantly higher during a long-term follow-up period in patients with CKD and in patients on chronic dialysis. Nevertheless, due to a high level of heterogeneity observed among several subgroups analyzed, randomized trials are required to completely solve this issue.

## 1 Introduction

Several recently published studies showed a rise in the number of patients suffering from Type 2 diabetes mellitus (T2DM) which could later be complicated by chronic kidney diseases (CKD), thus affecting approximately 13% of the population in the United States.^[1]^ Cardiovascular diseases (CVDs) are considered to be responsible for the significant increase in morbidity and mortality among patients suffering from an early or a late stage of CKD.^[2,3]^ Even in patients with end-stage renal disease (ESRD) undergoing chronic dialysis, CVDs were considered to be responsible for up to 44% of all-cause mortality.^[4]^

CKD is expected to rise rapidly in the next decade, but however, the impact of coronary artery bypass surgery (CABG) and percutaneous coronary intervention (PCI) on mortality in patients with CKD and in patients on chronic dialysis is still not very clear. For example, the analysis from a previously published study including patients from CKD cohorts showed CABG to favor patients with long-term mortality compared to PCI.^[5]^ However, the recently published study by Bangalore et al^[6]^ showed CABG to be associated with a significantly higher rate of death during a short-term follow-up period, while it was associated with a similar mortality rate in the long term, when compared to PCI. Therefore, in order to solve this issue, we aimed to show the impact of CABG and PCI on mortality in patients with CKD and in patients on chronic dialysis using a large number of patients obtained from CKD cohorts.

## 2 Methods

### 2.1 Data sources and search strategy

The Cochrane library, PubMed, EMBASE, and Medline databases were searched for studies comparing CABG with PCI in patients with CKD and in patients on chronic dialysis by typing the words or phrases “coronary artery bypass surgery and percutaneous coronary intervention and chronic kidney disease or dialysis.” The abbreviations “CABG, PCI, and CKD” were also used. To further enhance this search, the term “chronic kidney disease” was replaced by the terms “chronic renal disease or chronic kidney injury.” In addition, reference lists of relevant studies were also checked for suitable articles. Only English articles published as from the year 2002 were considered during this search process.

### 2.2 Inclusion and exclusion criteria

Studies were included if:

- They were observational studies (because all CKD cohorts were observational cohorts).
- They consisted only of patients with CKD (any stage was eligible).
- They compared CABG with PCI.
- They reported mortality and/or other adverse clinical outcomes as their endpoints during any follow-up period.
- They were published as from the year 2002.

Studies were excluded if:

- They were randomized controlled trials (RCTs) involving data from non-CKD cohorts, case studies, or meta-analyses.
- They did not compare CABG with PCI, but instead, reported the outcomes separately, that is, CABG and PCI were not compared, but these studies showed outcomes reported with CABG or PCI separately.
- They did not include patients with CKD or patients on chronic dialysis.
- Mortality was not reported among their clinical endpoints.
- They were published before the year 2002.
- They were duplicates.

### 2.3 Types of participants

All the participants included in this study were patients at different stages of CKD (early stage of CKD, mild, moderate, or severe CKD), patients with ESRD or even patients on chronic dialysis who underwent revascularization by either CABG or PCI. Different categories of patients with CKD involved are summarized in Table 1.

**Table 1:**
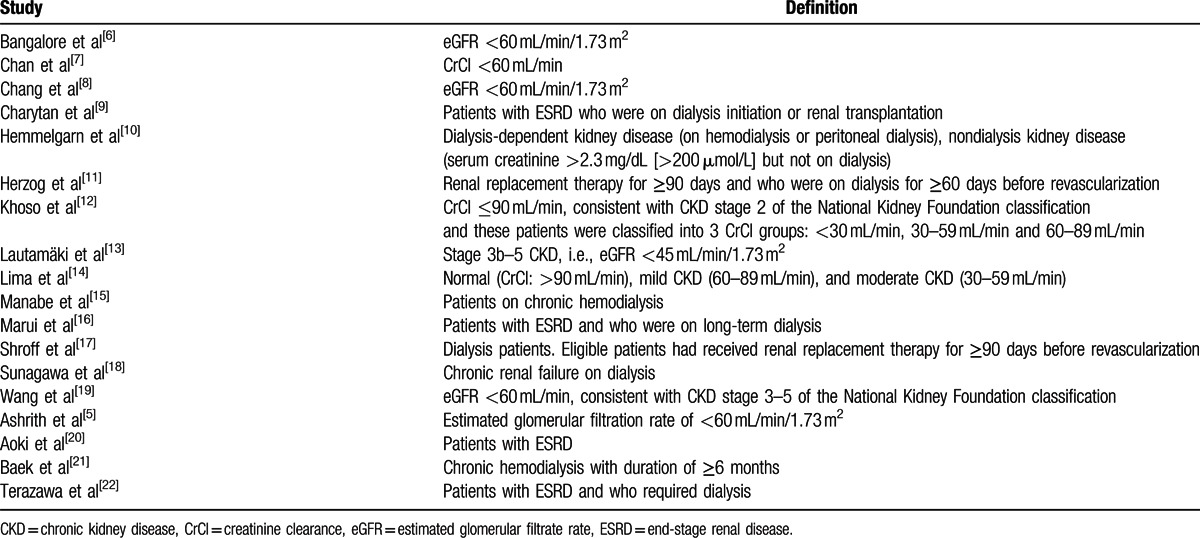
Definitions of chronic kidney diseases reported in the different cohorts involved.

### 2.4 Outcomes and follow-up periods

The adverse clinical outcomes included:

- Mortality (all-cause mortality)
- Myocardial infarction (MI)
- Stroke
- Repeated revascularization (involving target vessel revascularization and target lesion revascularization)
- Major adverse events (MAEs) consisting of major adverse cardiac events (MACEs), major adverse cardiovascular, and cerebrovascular events (MACCEs) and composite endpoints which involved death, MI, stroke, and repeated revascularization.

Follow-up periods included:

In-hospital follow up: mortality reported during the hospital stay.
Short-term follow up: mortality reported during a period of less than 1 year. In this current meta-analysis, most of the studies had a short-term follow-up period of 1 month.
Long-term follow up: adverse outcomes reported at or after a period of 1 year.

Table 2 lists the adverse clinical outcomes and corresponding follow up periods of the studies included.

**Table 2:**
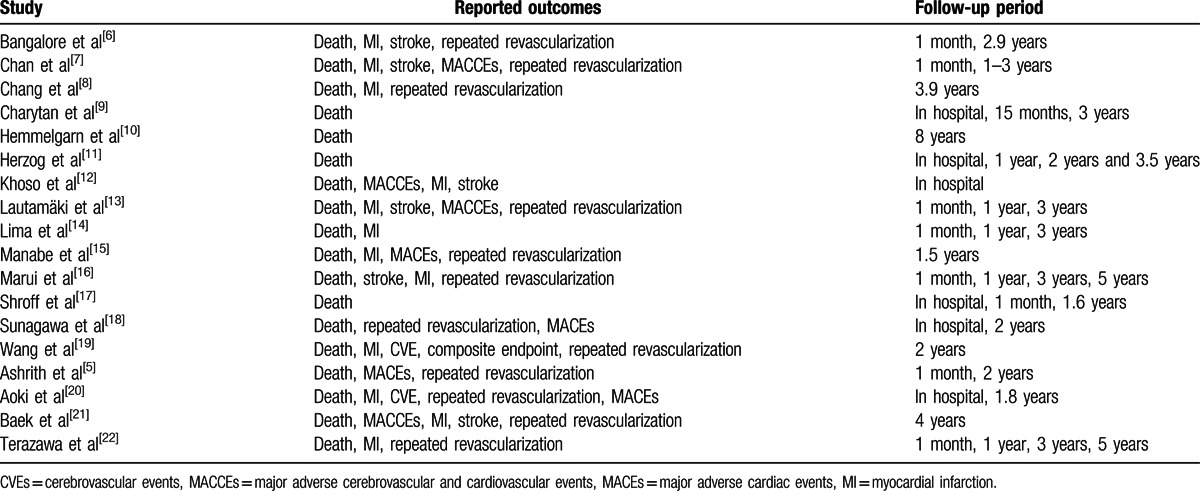
Reported outcomes among the included studies.

### 2.5 Data extraction and review

Two authors (PKB and AB) independently reviewed the data included in this systematic review and meta-analysis. Information regarding author names, year of publications, types of patients involved, total number of patients classified in the CABG and PCI groups respectively, number of patients on chronic dialysis, reported adverse clinical outcomes, causes of mortality among the patients and the follow-up periods were systematically extracted. If the authors disagreed about including certain studies or data, or could not reach a decision whether to accept or reject a study, disagreements were discussed carefully between the authors and a final decision was made. However, if the authors could not reach a consensus, disagreements were resolved by the third author (MHC).

### 2.6 Methodological quality and statistical analysis

The Preferred Reporting Items for Systematic Reviews and Meta-Analyses statement (PRISMA) was considered for this meta-analysis.^[23]^ Heterogeneity across the subgroups was carefully assessed using the Cochrane Q-statistic and the *I*^2^-statistic tests, respectively. For the Q-statistic test, a *P*-value ≤0.05 was considered statistically significant while a *P*-value greater than 0.05 was considered statistically insignificant. An *I*^2^ value of 0% was considered to be associated with a low level of heterogeneity whereas a larger value of *I*^2^ was associated with an increased heterogeneity. If *I*^2^ was less than 50%, a fixed effect model was used during the statistical analysis and if *I*^2^ was more than 50%, a random effect model was used. Odds ratios (ORs) with 95% confidence intervals (CIs) were calculated for categorical variables and the pooled analyses were performed with RevMan 5.3 software.

### 2.7 Ethics approval and patients consent

Ethical approval and patient consents were not indicated for systematic reviews and meta-analyses.

## 3 Results

### 3.1 Study selection and the general features of the included studies

**Figure 1:**
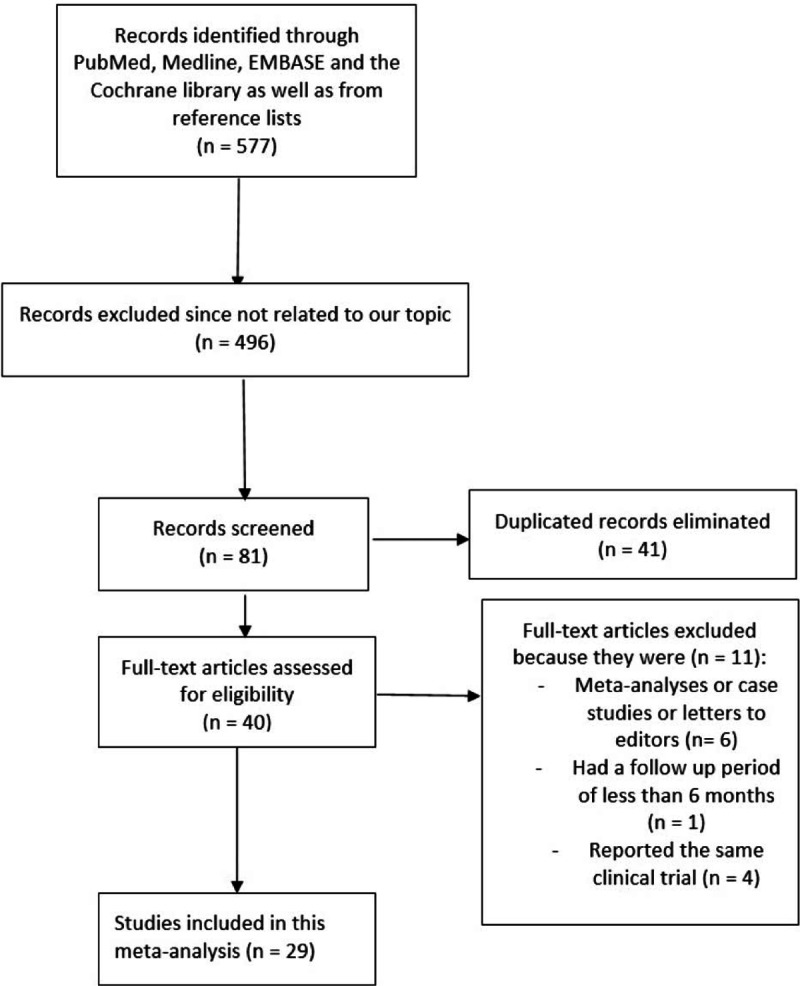
Flow diagram representing the study selection.

A total number of 1618 articles were obtained from electronic databases. A further 17 articles were obtained from the reference lists of highly relevant studies. One thousand six hundred five articles were eliminated since they were either not related to the topic of this research, or they were duplicates. Thirty full-text articles were assessed for eligibility. In the beginning, 5 articles were eliminated because they were meta-analyses and case studies. Another 5 articles were eliminated since they were published before the year 2002. Because non-CKD cohorts including randomized patients were not considered relevant in this study, 2 trials were further eliminated. Finally, 18 studies that satisfied all the inclusion and exclusion criteria of this study were selected. Figure 1 represents the flow diagram for study selection.

A total number of 69,456 patients (40,217 patients involving PCI and 29,239 patients involving CABG) were included in this analysis. The general features of the studies included are listed in Table 3.

**Table 3:**
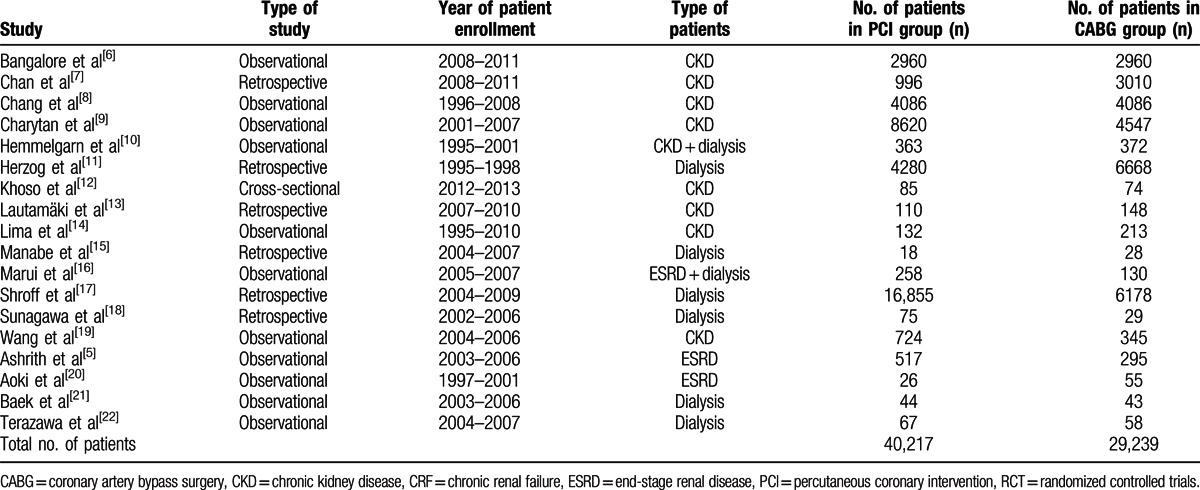
General features of the included studies.

The percentage of patients undergoing chronic dialysis, and the types of dialysis involved along with their corresponding duration period are listed in Table 4.

**Table 4:**
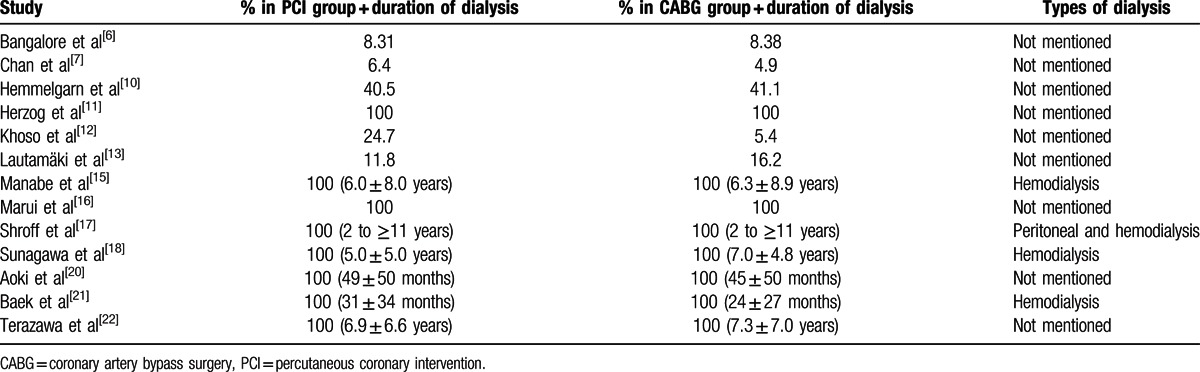
Percentage of patients undergoing dialysis.

Only 4 studies reported the types of dialysis undergone. Hemodialysis was reported in three studies whereas 1 study included both hemodialysis and peritoneal dialysis. The mean duration of dialysis ranged from 2 to more than 11 years. Further details are listed in Table 4.

### 3.2 Baseline characteristics

The baseline characteristics of the studies included in this meta-analysis are summarized in Table 5.

**Table 5:**
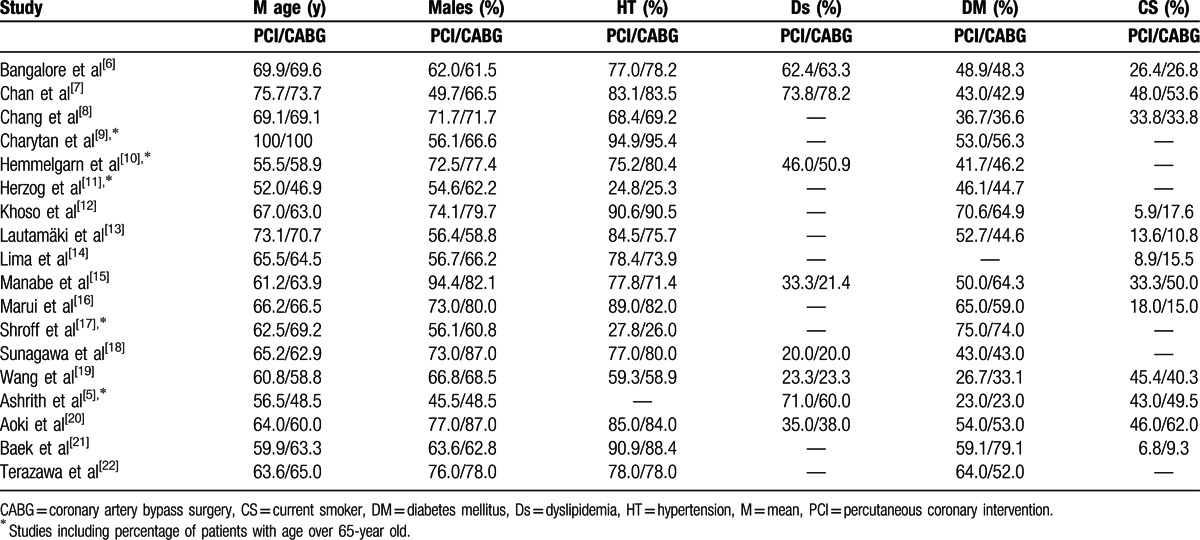
Baseline features of the studies included in this meta-analysis.

According to the baseline features, there were no significant differences between the 2 groups of patients (patients involved in the PCI group and patients involved in the CABG group).

### 3.3 Causes of mortality

All-cause death was considered as the primary endpoint in this analysis. However, the exact causes of death were also reported in certain studies.

Causes of death included:

Cardiac death
Death due to respiratory diseases
Death due to kidney disease/withdrawal from dialysis
Death due to infection
Death due to neoplasm
Other/unknown causes of death

Causes of cardiac death included:

Death due to heart failure
Death due to ischemic heart diseases
Sudden cardiac death
Valvular heart disease
Death due to cardiovascular diseases

The causes of death are listed in Table 6.

**Table 6:**
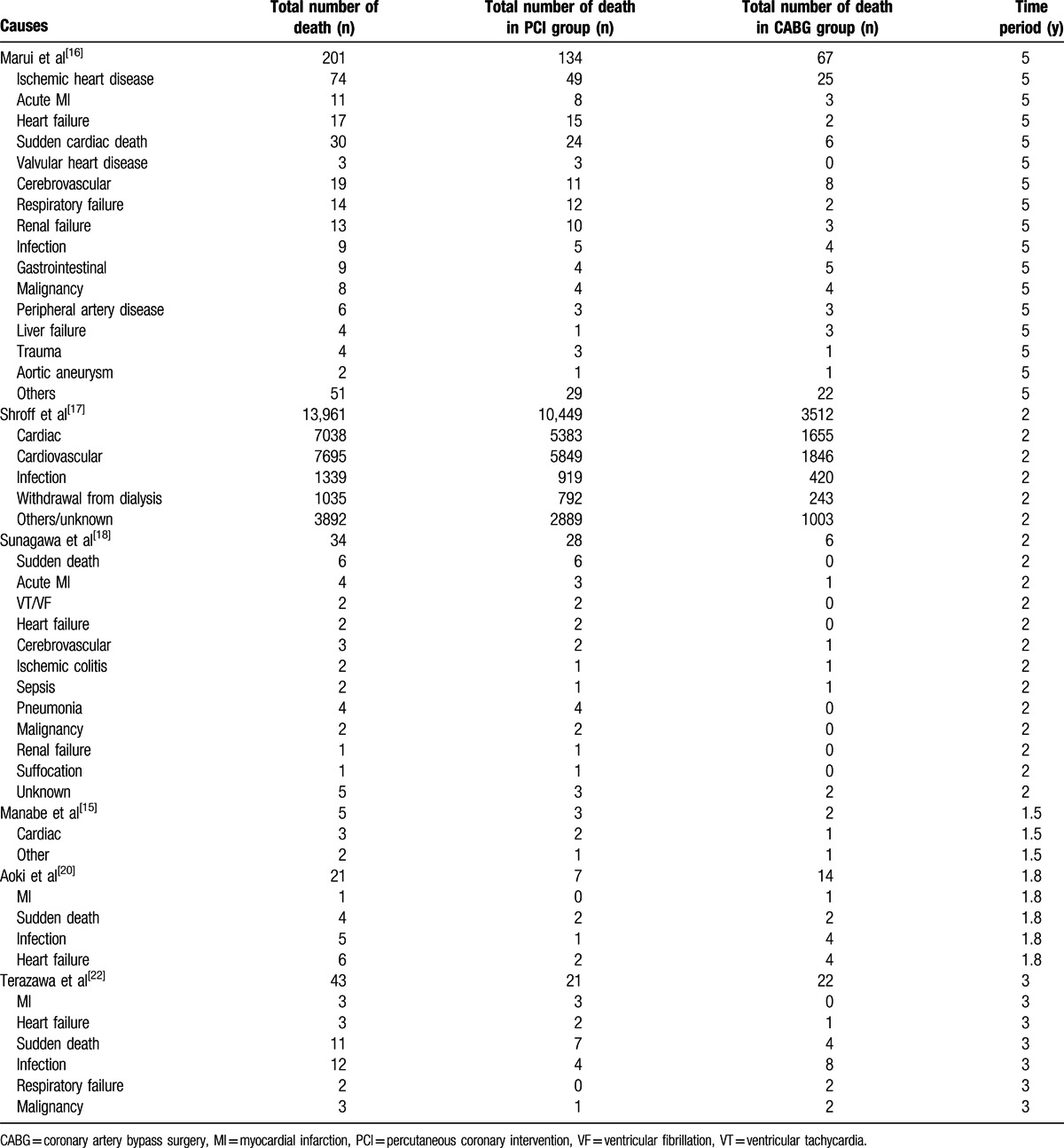
Causes of mortality in reported studies.

### 3.4 Mortality in patients with CKD

In this current analysis, patients with CKD, patients with ESRD and patients on chronic dialysis were separately analyzed for mortality.

**Figure 2:**
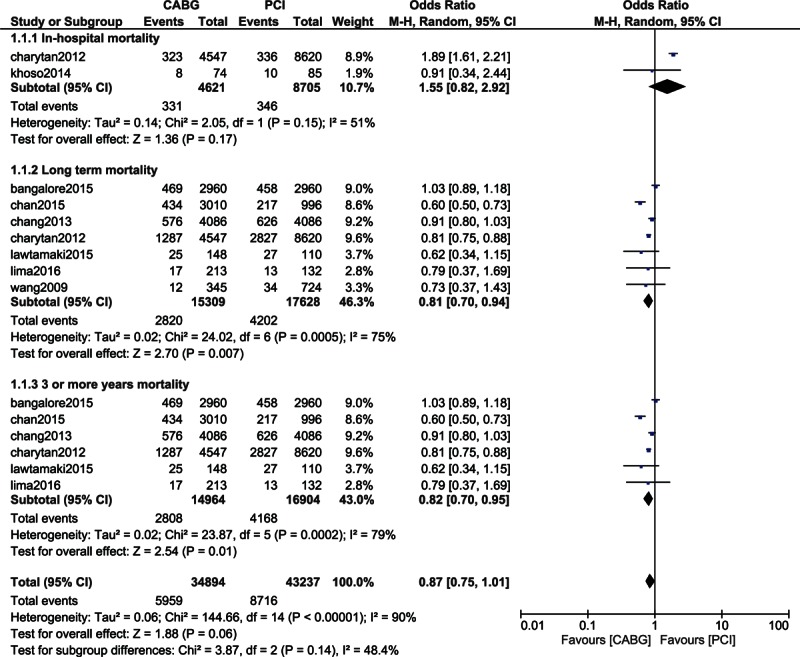
Mortality associated with CABG and PCI in patients with chronic kidney diseases.

During the in-hospital follow-up period, whereby 4621 patients from the CABG group and 8701 patients from the PCI group were analyzed, mortality favored PCI with OR: 1.55, 95% CI: 0.82–2.92; *P* = 0.17 (7.16% vs 3.97% in the CABG and PCI group, respectively) in these patients with CKD. However, the result was not statistically significant. This result is shown in Fig. 2.

**Figure 3:**
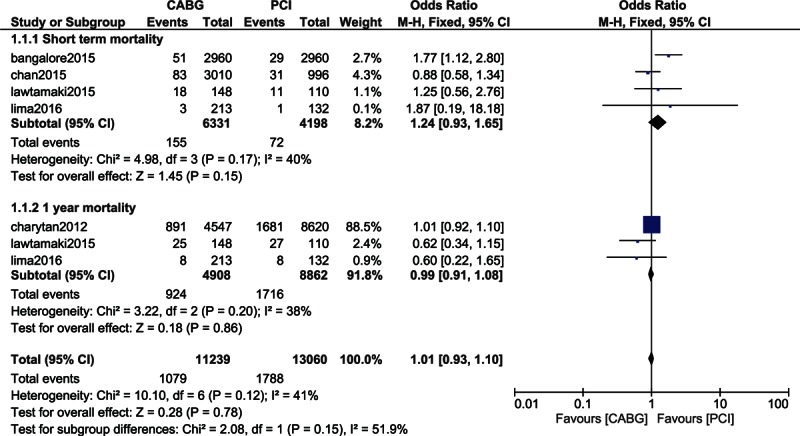
Mortality associated with CABG and PCI in patients with chronic kidney diseases.

When the short-term (1 month) mortality was analyzed in these patients with CKD, mortality again insignificantly favored PCI with OR: 1.24, 95% CI: 0.93–1.65; *P* = 0.15 among the 10,529 patients analyzed (2.45% vs 1.72%). This result is shown in Fig. 3.

Mortality at 1 year was not significantly different between CABG and PCI with OR: 0.99, 95% CI: 0.91–1.08; *P* = 0.86 (18.8% vs 19.4%) among the 13,770 patients analyzed. This result is represented in Fig. 3.

Long-term mortality (including a follow-up period of ≥1 year) significantly favored CABG with OR: 0.81, 95% CI: 0.70–0.94; *P* = 0.007 (18.4% vs 23.8%) among the 15,309 and 17,628 patients with CKD analyzed from the CABG and PCI groups, respectively. The result showing the long-term mortality is represented in Fig. 2.

When patients with CKD were analyzed for a longer period of time (3 or more years), mortality significantly favored CABG with OR: 0.82, 95% CI: 0.70–0.95; *P* = 0.01 (18.8% vs 24.7%) among the 31,868 patients analyzed. This result is illustrated in Fig. 2.

A low level of heterogeneity was observed among the subgroups analyzing short-term and 1-year mortality whereas an increased level of heterogeneity was observed among the subgroups analyzing long-term mortality in these patients with CKD.

### 3.5 Adverse clinical outcomes in patients with CKD

**Figure 4:**
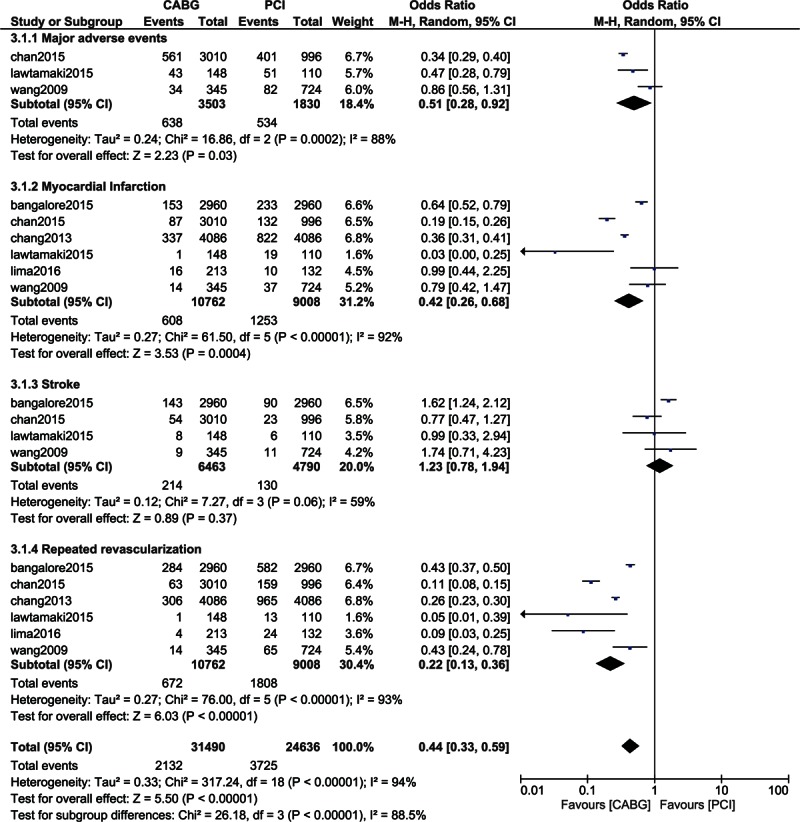
Analysis of the adverse clinical outcomes reported in patients with chronic kidney diseases.

When the other long-term adverse outcomes were analyzed in patients with CKD, MAEs, and MI significantly favored CABG with OR: 0.51, 95% CI: 0.28–0.92; *P* = 0.03 (18.2% vs 29.2%) and OR: 0.42, 95% CI: 0.26–0.68; *P* = 0.0004 (5.65% vs 13.9%), respectively. Stroke favored PCI with OR: 1.23, 95% CI: 0.78–1.94; *P* = 0.37 (3.31% vs 2.71%). However, this result was not statistically significant. Moreover, CABG was associated with a significantly lower rate of repeated revascularization with OR: 0.22, 95% CI: 0.13–0.36; *P* < 0.00001 (6.24% vs 20.1%). However, a high level of heterogeneity. Results showing the long-term adverse clinical outcomes between CABG and PCI in patients with CKD are represented in Fig. 4.

### 3.6 Mortality in patients on chronic dialysis

**Figure 5:**
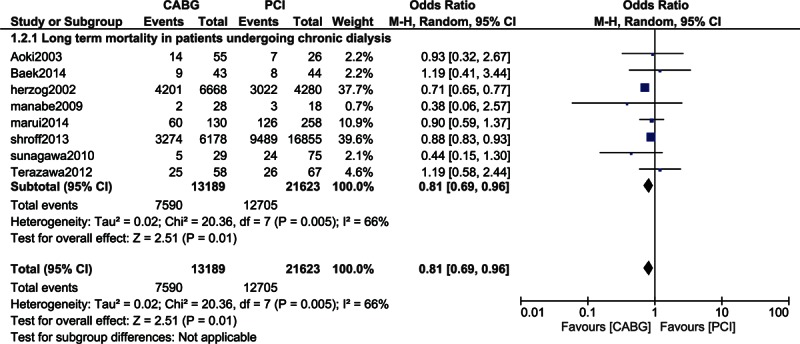
Mortality in patients on chronic dialysis.

When patients undergoing chronic dialysis were analyzed, long-term mortality significantly favored CABG with OR: 0.81, 95% CI: 0.69–0.96; *P* = 0.01 (57.5% vs 58.8%) among the 34,812 patients analyzed. However, an increased level of heterogeneity was observed in this subgroup of patients (Fig. 5).

### 3.7 Adverse outcomes in patients on chronic dialysis

**Figure 6:**
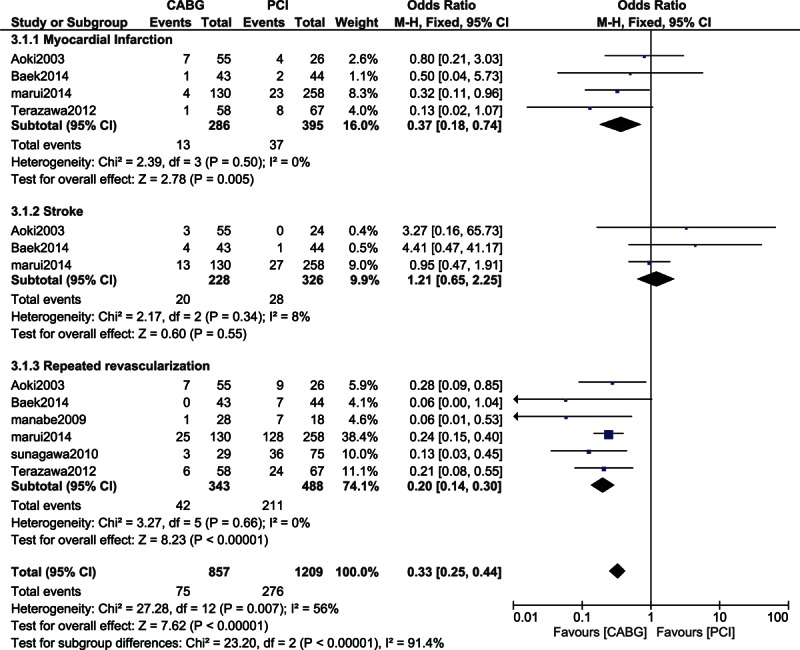
Analysis of the adverse clinical outcomes reported in patients on chronic dialysis.

Moreover, when the other adverse clinical outcomes were specifically analyzed in patients with chronic dialysis, MI and repeated revascularization significantly favored CABG with OR: 0.37, 95% CI: 0.18–0.74; *P* = 0.005 (4.54% vs 9.37%) and OR: 0.20, 95% CI: 0.14–0.30; *P* < 0.00001 (8.75% vs 22.8%), respectively. However, even if stroke favored PCI with OR: 1.21, 95% CI: 0.65–2.25; *P* = 0.55 (8.77% vs 8.59%), this result was not statistically significant. A low level of heterogeneity was observed when analyzing the adverse clinical outcomes between CABG and PCI in patients on chronic dialysis (Fig. 6).

### 3.8 Mortality among patients with ESRD

**Figure 7:**
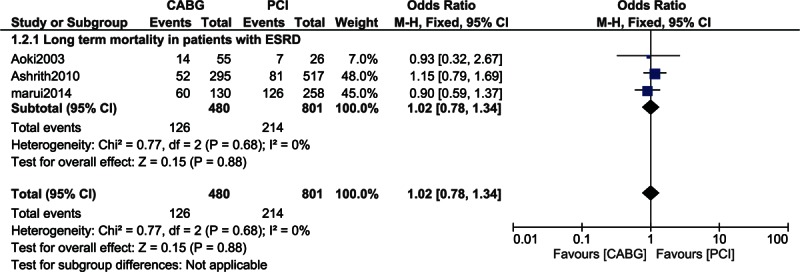
Mortality in patients with end-stage renal diseases.

Another subgroup comparing the long-term mortality associated with CABG and PCI in patients with ESRD with or without dialysis showed both revascularization strategies to be associated with a similar mortality rate with OR: 1.02, 95% CI: 0.78–1.34; *P* = 0.88 (26.3% vs 26.7%). This result which involved a low level of heterogeneity is represented in Fig. 7.

### 3.9 Analysis of the causes of mortality

**Figure 8:**
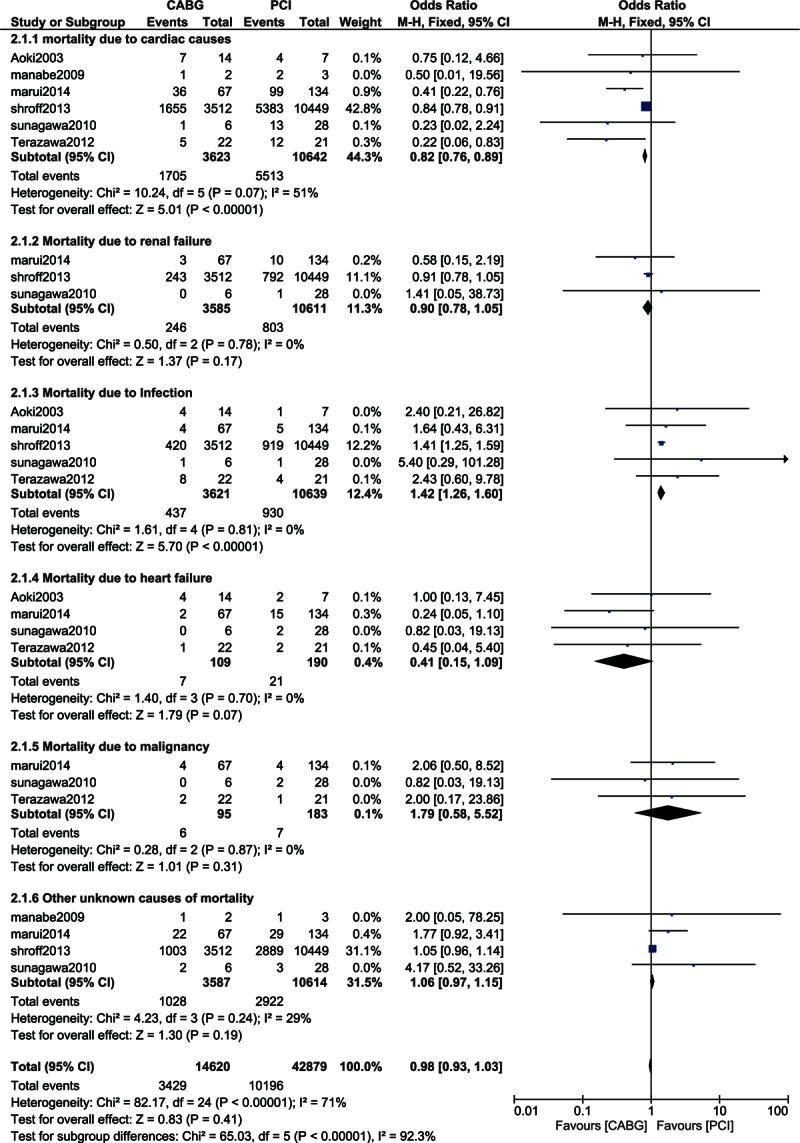
Analysis of the causes of mortality.

The causes of mortality were also analyzed. Mortality due to cardiac causes was significantly lower in the CABG group with OR: 0.82, 95% CI: 0.76–0.89; *P* < 0.00001. Mortality due to renal and heart failure significantly favored CABG with OR: 0.90, 95% CI: 0.78–1.05; *P* = 0.17 and OR: 0.41, 95% CI: 0.15–1.09; *P* = 0.07, respectively. However, these results were not statistically significant. Moreover, mortality due to infection was significantly higher in the CABG group with OR: 1.42, 95% CI: 1.26–1.60; *P* < 0.00001. In addition, other unknown causes of mortality were similar between the CABG and PCI groups with OR: 1.06, 95% CI: 0.97–1.15; *P* = 0.19. These results are represented in Fig. 8.

## 4 Discussion

This study aimed to show the impact of CABG and PCI on mortality in patients with CKD and in patients on chronic dialysis. Results of this current analysis showed that in-hospital and short-term mortality favored PCI in these patients with CKD but these results were not statistically significant. Moreover, CABG and PCI were associated with a similar 1-year mortality rate in these patients with CKD. However, long-term mortality significantly favored CABG. When patients on chronic dialysis were analyzed, the long-term mortality again significantly favored CABG. However, in the subgroup of patients with ESRD, CABG and PCI were associated with a similar rate of long-term mortality.

Reasons contributing to such a result could be related to the fact that higher rates of restenosis and incomplete revascularization were associated with PCI. Another possible mechanism explaining a higher rate of long-term mortality observed among patients who were revascularized by PCI could be the increased risk of contrast induced acute nephropathy which was also associated with a greater risk of adverse clinical events after PCI. Use of the left internal mammary artery graft in patients who underwent CABG could also be among the reasons why CABG was associated with better outcomes compared to PCI.

The meta-analysis published by Chen et al,^[24]^ including a total number of 38,740 patients showed that CABG was associated with a higher short-term mortality (OR: 0.55, 95% CI: 0.41–0.73; *P* < 0.01) but however, a higher long-term mortality was associated with PCI (OR: 1.29, 95% CI: 1.23–1.35; *P* < 0.01). Their results were almost similar to the results of this current study. In addition, the review explaining the management of coronary artery disease in patients with CKD and ESRD suggested that CABG was associated with an increased long-term survival as well as a reduced repeated revascularization when compared to PCI^[25]^ again supporting the results of this analysis during the long-term follow up. However, in this current study, a similar rate of mortality was observed among patients with CKD at 1-year follow up and in patients with ESRD, respectively. Another study demonstrating the impact of CKD on long-term outcomes showed CABG to be associated with a lower long-term mortality compared to PCI^[14]^ in a subgroup of patients with T2DM. However, results from this current analysis did not include any separate subgroup of T2DM, but instead, a mixed population of patients with and without T2DM was included.

While assessing the economic attractiveness of coronary artery revascularization in patients with CKD, the author predicted that CABG was an economically attractive alternative compared to PCI or medical therapy for all CKD patients with 2 vessels coronary artery disease.^[26]^

Bangalore et al performed a separate propensity score matching specifically on 486 patients who underwent dialysis. Similar to the results of this current study, their results which involved data from registries, also showed CABG to be associated with a significantly lower long-term mortality rate compared to PCI (39.1% vs 54.3% with HR 2.02, 95% CI: 1.40–2.93; *P* = 0.0002) in these patients on chronic dialysis.^[6]^

Since previously published trials involved data mainly from non-CKD cohorts, the Arterial Revascularization Therapies Study (ARTS) trial showed a similar mortality rate between CABG and PCI in patients with CKD at 3 years (OR: 0.93, 95% CI: 0.54–1.60; *P* = 0.97),^[27]^ and the authors stated that even at 5 years, the rate of death was not statistically significant between these 2 groups (12.3% in the CABG group vs 14.5% in the PCI group)^[28]^ which was completely different from the results of this current study which included data from CKD cohorts and showed a significantly higher mortality associated with PCI compared to CABG during the long-term follow up (>1 year).

Other studies which reported different results from this current meta-analysis included the clinical update by Cai et al^[29]^ reviewing coronary artery disease in patients with CKD. The authors concluded that compared to PCI, CABG was associated with significant perioperative morbidity and mortality. In addition, the study by Wang et al^[19]^ showed a similar rate of mortality, MI, and cerebrovascular events between CABG and PCI in patients with CKD. However, their study had several potential limitations which were not identical to this current study. It was a single-study based non-RCT that compared 2 vessel diseases with 3 vessel diseases in patients with CKD.

This current analysis involved patients with mild or moderate CKD, patients with ESRD and patients on chronic dialysis. In the subgroups reporting a similar mortality rate between CABG and PCI, for example, in patients with CKD at 1 year follow up or in patients with ESRD, the SYNergy between PCI with TAXUS™ and Cardiac Surgery (SYNTAX) score could be used to decide which revascularization procedure would be beneficial.^[30]^ The SYNTAX angiographic grading system has previously been used alone to identify potential risk for revascularization. A higher SYNTAX score is indicative of more complex disease and is hypothesized to represent a bigger therapeutic challenge and associated with a poor prognosis. Hence, representing greatest risk to patients undergoing PCI.

## 5 Novelty

This study is new in several ways. It is among the first meta-analyses comparing CABG with PCI in patients with CKD, ESRD, and patients on chronic dialysis. Moreover, the different causes of mortality were also compared between CABG and PCI (including different cardiac causes of mortality, mortality due to infection, mortality due to renal failure, mortality due to malignancy, and other unknown causes). Also, long-term adverse clinical outcomes such as MAEs, MI, stroke, and repeated revascularization were also assessed. By representing all these results in 1 study, this study projects a new aspect showing the impact of CABG and PCI on mortality in patients with different stages of CKD and in patients on chronic dialysis.

## 6 Limitations

Similar to other studies, this study also has several limitations. First of all, due to a limited number of patients, this analysis might not provide robust results. Only patients from observational studies were included. Since data from observational studies are not as good as data from randomized trials, involving data from observational cohorts might not provide great results. However, because there was no randomized cohort comparing CABG and PCI in patients with CKD, we had no other choice than including data only from observational cohorts. Moreover, a high level of heterogeneity was observed among the subgroups analyzing mortality and other adverse outcomes representing a major limitation of this study. This current meta-analysis involved observational studies published in or after the year 2012. Selection bias and publication bias could have contributed to the high level of heterogeneity. In addition, this analysis which involved only articles published in English could be affected by language bias.

## 7 Conclusion

In patients with CKD, the impact of CABG on the short-term mortality was insignificantly higher compared to PCI whereas at 1 year, a similar impact was observed. However, the impact of PCI on mortality was significantly higher during a long-term follow-up period in patients with CKD and in patients on chronic dialysis. Nevertheless, due to a high level of heterogeneity observed among several subgroups analyzed, randomized trials are required to completely solve this issue.
